# Mysm1 is required for interferon regulatory factor expression in maintaining HSC quiescence and thymocyte development

**DOI:** 10.1038/cddis.2016.162

**Published:** 2016-06-09

**Authors:** X F Huang, V Nandakumar, G Tumurkhuu, T Wang, X jiang, B Hong, L Jones, H Won, H Yoshii, K Ozato, A Masumi, S-Y Chen

**Affiliations:** 1 Department of Molecular Microbiology and Immunology, Norris Comprehensive Cancer Center, Keck School of Medicine, University of Southern California, Los Angeles, CA, USA; 2 Laboratory of Molecular Growth Regulation, Program in Genomics of Differentiation, National Institute of Child Health and Human Development, National Institutes of Health, Bethesda, MD, USA; 3 Department of Safety Research on Blood and Biological Products, National Institute of Infectious Diseases, Tokyo, Japan

## Abstract

Mysm1^−/−^ mice have severely decreased cellularity in hematopoietic organs. We previously revealed that Mysm1 knockout impairs self-renewal and lineage reconstitution of HSCs by abolishing the recruitment of key transcriptional factors to the Gfi-1 locus, an intrinsic regulator of HSC function. The present study further defines a large LSKs in >8-week-old Mysm1^−/−^ mice that exhibit increased proliferation and reduced cell lineage differentiation compared with those of WT LSKs. We found that IRF2 and IRF8, which are important for HSC homeostasis and commitment as transcription repressors, were expressed at lower levels in Mysm1^−/−^ HSCs, and Mysm1 enhanced function of the IRF2 and IRF8 promoters, suggesting that Mysm1 governs the IRFs for HSC homeostasis. We further found that the lower expressions of IRF2 and IRF8 led to an enhanced transcription of p53 in Mysm1^−/−^ HSCs, which was recently defined to have an important role in mediating Mysm1^−/−^-associated defects. The study also revealed that Mysm1^−/−^ thymocytes exhibited lower IRF2 expression, but had higher Sca1 expression, which has a role in mediating thymocyte death. Furthermore, we found that the thymocytes from B16 melanoma-bearing mice, which display severe thymus atrophy at late tumor stages, exhibited reduced Mysm1 and IRF2 expression but enhanced Sca1 expression, suggesting that tumors may downregulate Mysm1 and IRF2 for thymic T-cell elimination.

Histones ubiquitination is one of the major epigenetic modifications occurring on histone tails. Among the histone octamers, H2A is the most highly ubiquitinated, and PRC1 is the main H2A ubiquitin ligase.^1, 2^ Once PRC1 is localized to the chromatin, its core components Ring1B and Bmi1 ubiquitinate histone H2A at lysine 119,^1, 2, 3^ which contributes to transcriptional repression by inhibiting transcription initiation^4^ or by restraining RNA pol II-mediated transcription elongation.^5^ While H2A ubiquitinations have important roles in repressing gene transcription, H2A-specific deubiquitinations are required for gene activation, which are involved in regulating HOX gene silencing, X chromosome inactivation, cell-cycle progression, DNA damage repair, and liver regeneration.^4, 6, 7, 8, 9^

The Mysm1 is a recently identified H2ADUB that functions as an activator via the removal of a polyubiquitin chain at lysine 119 of histone H2A.^10^ Mysm1-mediated H2A deubiquitination is important in the activation of several transcriptional events, including AR-regulated target gene transcription in prostate cancer cells.^10^ We recently generated an Mysm1-deficient mouse line (*Mysm1*^−/−^), which resembles the clinical manifestation of Mysm1 mutation in the reported human cases,^11, 12^ and exhibits overall decreased cellularity in all of the blood organs.^13, 14, 15, 16, 17^ Our previous work revealed critical roles of Mysm1-mediated H2A deubiquitination in HSC differentiation,^16^ NK cell maturation,^14^ and B-cell and dendritic cell (DC) commitments.^13, 18^ We found that Mysm1^−/−^ impairs self-renewal and lineage reconstitution of HSC by abolishing recruitment of key transcriptional factors to the Gfi-1 locus.^16^ In the present study, we further investigated the role of Mysm1 in the maintenance of cell lineages by systematically analyzing the impact of Mysm1 deficiency on T-cell lineages. We found that Mysm1 is required for the expression of IRFs in maintaining HSC quiescence and thymocyte development.

## Results

### Defective T-cell development in Mysm1^−/−^ mice

Mysm1^−/−^ mice exhibit overall decreased cellularity in hematopoietic organs. Despite a slightly increased frequency of T lymphocytes, Mysm1^−/−^ mice exhibit a drastic reduction in CD4^+^ and CD8^+^ T-cell numbers in secondary lymphoid tissues, including spleen and inguinal LNs (Supplementary Figure 1A and B), implying a severe T cell-associated lymphocytopenia in Mysm1^−/−^ mice.

T cells originate from HSCs in BM. Lymphoid progenitors from HSCs populate thymus via cell division,^19^ developing from the double-negative (CD4^−^CD8^−^) and double-positive (CD4^+^CD8^+^) populations, and maturing to single-positive (CD4^+^CD8^−^ or CD4^−^CD8^+^) thymocytes before release into peripheral tissues.^19^ However, the 8-week-old Mysm1^−/−^ mice were found to have severely reduced thymus sizes (~1/6–1/5 of the WT thymus size) (Supplementary Figure 1C). The absolute numbers of live thymocyte at all four stages were dramatically reduced (Supplementary Figure 1E*, lower*), albeit with slight changes in frequencies (Supplementary Figure 1D and 1E*, top*). The number of ETPs, the earliest progenitors of T cells in thymus with the phenotype Lin^−^CD4^−^CD8^−^CD44^+^CD25^−^cKit^+^,^19^ was also much lower in Mysm1^−/−^ thymi than that in WT thymi (Supplementary Figure 1F).

Common lymphoid progenitors (CLPs) are defined as Lin^−^Sca1^int^cKit^int^IL-7R^+^ and represent an early progenitor committed to lymphocyte differentiation in BM.^20^ Mysm1^−/−^ mice (~8 weeks old) had approximately a fourfold decrease in total BM cell numbers (Supplementary Figure 1G), and a greater than twofold decrease in CLP frequency (Supplementary Figure 1H), which led to very few CLPs in Mysm1^−/−^ BM. These results demonstrate that in all of the analyzed T-cell compartments, Mysm1^−/−^ mice had drastically decreased T-cell numbers.

### HSC defects in T-cell lymphopoiesis in Mysm1^−/−^ mice

**Figure 1:**
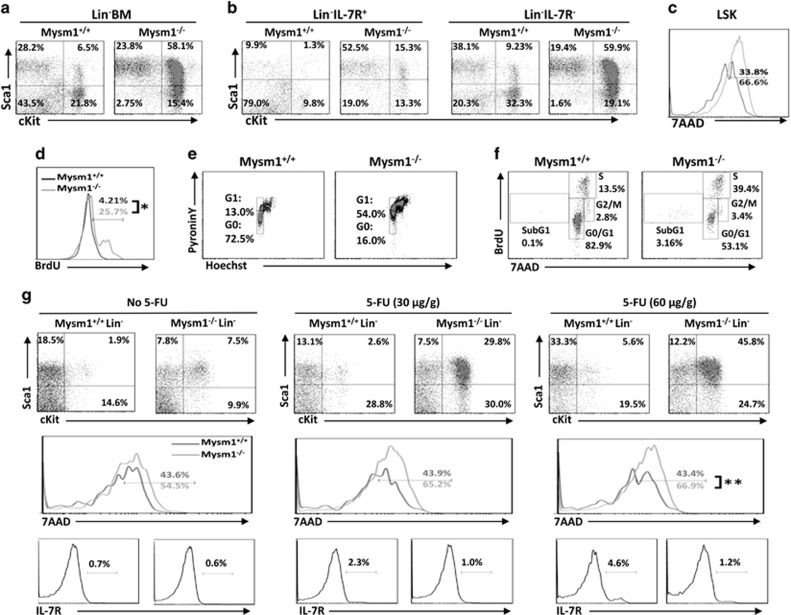
HSC differentiation in Mysm1^−/−^ mice. (**a**) BM cells from 8- to 10-week-old WT or Mysm1^−/−^ mice were analyzed by FACS for the frequency of LSK; (**b**) for the frequencies of LSK IL-7R^+^ and LSK IL-7R^−^; and (**c**) for the death propensity of LSK by 7AAD staining. The data shown are from one of three independent experiments (*n*=4). (**d**) Eight- to ten-week-old WT or Mysm1^−/−^ mice received 1 mg of BrdU i.p. injection daily for 5 days. Incorporation of BrdU was analyzed by FACS to determine the proliferation of LSKs (*n*=4). (**e**) BM cells from 8- to 10-week-old WT or Mysm1^−/−^ mice were analyzed by Hoechst 33258/Pyronin Y staining for cell-cycle phases of LSKs (*n*=4). (**f**) Eight- to ten-week-old WT or Mysm1^−/−^ mice were injected with 2 mg of BrdU. One hour later, the mice were euthanized and BM cells were analyzed for cell-cycle kinetics of LSKs (*n*=4). (**g**) Five- to six-week-old WT or Mysm1^−/−^ mice received i.p. injection of 5-FU at 0, 30, or 60 *μ*g/g of body weight. Forty-eight hours post injection, BM cells were harvested from the euthanized mice and analyzed for frequencies (*top*) and cell death (*middle*) of LSKs, and for expression of IL-7R (*lower*) in Lin^−^ BM cells. Data are representative of three independent experiments. *P<0.01; **P<0.05

Our previous results showed that Mysm1 deficiency impairs HSCs by failing to recruit key transcriptional factors to Gfi-1. However, as the deletion of Mysm1 results in global changes in gene expression as demonstrated by a pilot microarray analysis,^16^ Mysm1 may also epigenetically regulate the differentiation of HSCs into T cells through Gfi1-independent mechanisms. Thus, BM cells from Mysm1^−/−^ mice were re-evaluated for HSC differentiation. Immunostaining followed by FACS analyses defined a large (>50%) LSK cell population in Mysm1^−/−^ mice (~8 weeks old). In WT mice, the corresponding LSK population was less than 10% (Figure 1a). This population was not observed in our previous study^16^ as it accumulates with age and only becomes apparent after ~8 weeks. While this observation has been reported by Nijnik *et al.*,^15^ it was not closely explored. This LSK population consisted of both IL-7R^+^ LSK and IL-7R^−^ LSK cells in Mysm1^−/−^ mice (Figure 1b), and there was a twofold increase in the propensity for cell death compared with WT LSKs (Figure 1c).

To determine how the LSK cells developed, an *in vivo* BrdU incorporation assay was performed, and the results showed that a significantly higher proportion of Mysm1^−/−^ LSKs had incorporated BrdU into the cellular DNAs 5 days after BrdU injection compared with that of WT LSKs (25.7% *versus* 4.2%, respectively) (Figure 1d). Pyronin/Hoechst staining was performed to measure the RNA/DNA content, which revealed that an elevated percentage of Mysm1^−/−^ LSKs had entered the G_1_ phase compared with WT LSKs (54% *versus* 13%, respectively) (Figure 1e). Moreover, the short-term BrdU labeling assay evaluating cell-cycle phase revealed that a higher frequency of Mysm1^−/−^ LSKs was at the S phase (~39.4% *versus* 13.5% of WT LSKs) (Figure 1f). Together, these results reveal the rapid proliferative ability of Mysm1^−/−^ LSKs. Proliferating HSCs are sensitive to the anti-proliferative agent 5-FU. We thereby used increasing dosages of 5-FU to treat 4- to 5-week-old Mysm1^−/−^ or WT mice when the LSK population was less obvious, and analyzed the mice 48 h later. The 5-FU treatment expanded the LSK populations in both WT and Mysm1^−/−^ mice. However, the LSK expansion in the Mysm1^−/−^ mice (from 7.5% to 29.8% or 45.8%) was noticeably greater than that in WT mice (from 1.9% to 2.6% or 5.6%) (Figure 1g, *top*). 5-FU treatment also led to a higher percentage of LSK death (from 54.5% to 66.9%) in Mysm1^−/−^ mice. However, the increase in death due to 5-FU treatment (1.2-fold) was far from the increase in LSK proliferation in Mysm1^−/−^ mice (4- to 6-fold) (Figure 1g, *middle*). This may explain why a much larger LSK population was observed in Mysm1^−/−^ BM.

**Figure 2:**
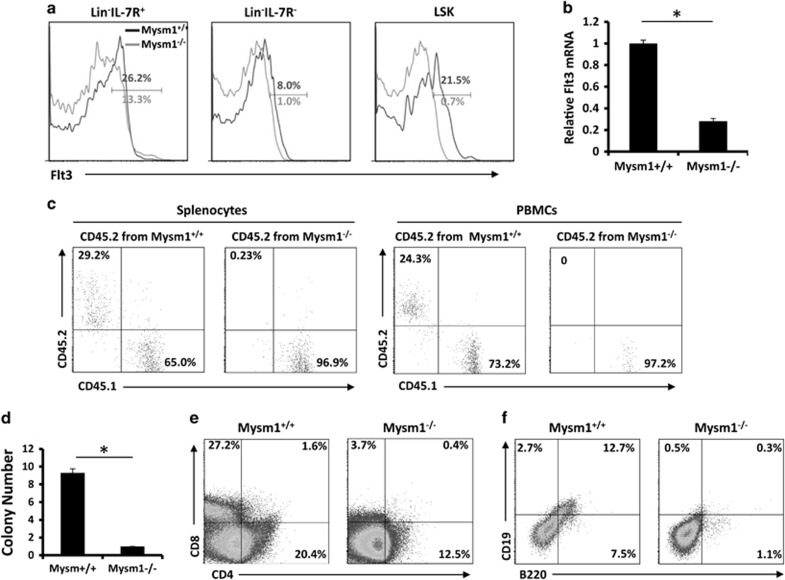
LSKs analyzed for T-cell lymphopoiesis in Mysm1^−/−^ mice. (**a**) FACS analysis of expression of Flt3 in Lin^−^ BM cells and LSKs (*n*=4). (**b**) Expression of Flt3 in Lin^−^ BM as determined by qPCR (*n*=4). Bar graphs show means of three experiments±S.D. *P<0.05. (**c**) LSKs (CD45.2) isolated from WT or Mysm1^−/−^ mice were mixed with WT BM cells (CD45.1) and i.v. injected into WT mice (CD45.1). Three weeks later, FACS analyzed splenocytes and PMBCs on differentiation of LSKs into T cells (*n*=4). (**d**) LSKs isolated from WT or Mysm1^−/−^ mice (*n*=4) were cultured on feeder cell line (OP-9DL) monolayers in IL-7 and Flt3L-supplemented media in 96-well plates for T-cell colony formation. (**e**) The cultured LSKs (*n*=4) on feeder cell monolayers were analyzed for expression of T-cell markers CD4 or CD8. (**f**) The cultured LSKs on feeder cell monolayers were analyzed for expression of B-cell markers CD19 and B220. Data are representative of three independent experiments

Although CLPs, defined as Lin^−^Sca1^int^cKit^int^IL-7R^+^, are the main progenitor for lymphopoiesis, prior studies have revealed that LSK, that is, Lin^−^Sca1^+^cKit^+^, in BM have the potential for lymphopoiesis based on their expression of IL-7R and Flt3.^21, 22^ The 5-FU treatment described above was found to result in a gradual increase in IL-7R expression in WT LSK cells, whereas Mysm1^−/−^ LSK cells maintained IL-7R expression at low levels (Figure 1g, *lower*). We further evaluated the expression of Flt3 in LSKs that signify the earliest lymphoid commitment step from HSC.^22^ Consistent with our previous report,^17^ Lin^−^ BM cells in Mysm1^−/−^ mice expressed lower levels of Flt3 relative to WT Lin^−^ BM cells regardless of IL-7R expression. Additionally, the observed LSK population in Mysm1^−/−^ BM largely lost Flt3 expression as revealed by FACS (Figure 2a) and qPCR (Figure 2b).

We next used competitive transplantation assays to study differentiation of the LSKs into T cells. Groups of lethally irradiated mice (CD45.1) received 1 × 10^5^ sorted LSKs from WT or Mysm1^*−/−*^ BM (CD45.2) along with 2 × 10^5^ competitor BM cells (CD45.1). Three to four weeks after transplantation, the percentage of WT or Mysm1^−/−^ T cells (CD45.2) was analyzed. It was revealed that the Mysm1^−/−^ LSKs markedly lost their ability to reconstitute T-cell lineages as determined from the PBMCs and spleens of recipient mice (Figure 2c). We further evaluated the potential of these LSKs to differentiate into T cells *in vitro* by incubating the sorted LSKs from WT or Mysm1^−/−^ mice on feeder cell (OP9-DL) monolayers in media supplemented with IL-7 and Flt3L for 2 weeks. Consistent with reduced IL-7R and Flt3 expression in Mysm1^−/−^ HSCs, a cell colony formation assay revealed that there were fewer colonies in Mysm1^−/−^ LSK cultures than in WT LSK cultures (Figure 2d). Immunostaining/FACS analysis showed that there were fewer cells from Mysm1^−/−^ LSK cultures on OP9-DL feeder monolayers that expressed T-cell markers (CD4 or CD8) (Figure 2e) and there were also fewer cells from the Mysm1^−/−^ LSK cultures on OP9 feeder monolayers that expressed B-cell markers (CD19 and B220) (Figure 2f) than those from WT LSK cultures. These data demonstrate that Mysm1 deficiency facilitates the rapid differentiation of HSCs into dysfunctional LSKs.

### Mysm1 deficiency reduces IRF2 and IRF8 expression in HSCs

**Figure 3:**
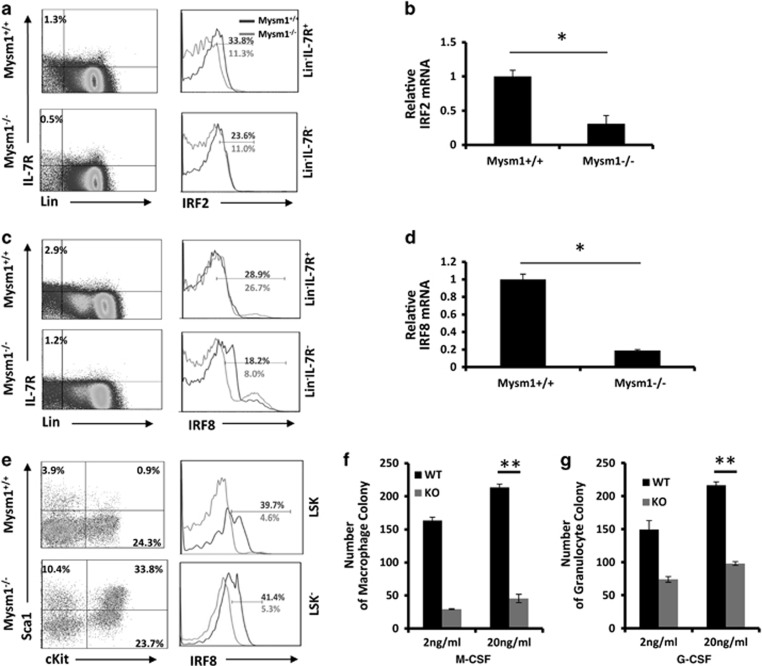
HSCs express lower levels of IRF2 and IRF8 in Mysm1^−/−^ mice. (a and **b**) BM cells from 8- to 10-week-old WT or Mysm1^−/−^ mice (*n*=4) were analyzed for expression of IRF2 in Lin^−^IL-7R^+^ or Lin^−^IL-7R^−^ HSCs by ICS (**a**) or qPCR (**b**). (**c** and **d**) BM cells from 8- to 10-week-old WT or Mysm1^−/−^ mice (*n*=4) were analyzed for expression of IRF8 in Lin^−^IL-7R^+^ or Lin^−^IL-7R^−^ HSCs by ICS (**c**) or qPCR (**d**). (**e**) BM cells from 8- to 10-week-old WT or Mysm1^−/−^ mice were analyzed for expression of IRF8 in Lin^−^Sca1^−^cKit^+^ HSCs including CMPs, GMPs, and MEPs by ICS (*n*=4). (**f**) Lin^−^ BM from 8- to 10-week-old WT or Mysm1^−/−^ mice (*n*=4) was cultured in M-CSF-supplemented methylcellulose media for macrophage colony formation. (**g**) Lin^−^ BM from 8- to 10-week-old WT or Mysm1^−/−^ mice (*n*=4) was cultured in G-CSF/IL-3-supplemented methylcellulose media for granulocyte colony formation. Data are representative of three independent experiments. Bar graphs show means of three experiments±S.D. *P<0.01, **P<0.05

IRF2 and IRF8 can function as transcription repressors to preserve self-renewal of HSCs,^23, 24, 25^ or to limit lineage differentiation.^26, 27^ Previous studies analyzing IRF2^−/−^ mice revealed that IRF2 preserves self-renewal and long-term lineage reconstitution of HSCs by limiting LSK generation.^23, 25^ Studies analyzing IRF8^−/−^ mice showed that IRF8 reduced cKit expression,^26^ and that IRF8^−/−^ mice enhanced granulocyte development in various lymphoid organs.^28, 29^ These studies prompted us to test whether Mysm1^−/−^ mice generate the dysfunctional LSK population that exhausts HSCs through IRF2 and/or IRF8 reduction. ICS revealed that IRF2 expression was significantly reduced in the whole Lin^−^ BM population in Mysm1^−/−^ mice regardless of IL-7R expression (Figure 3a). IRF8 downregulation was limited to Lin^−^IL-7R^−^ BM cells (Figure 3c), and was also prominent in both LSK and Lin^−^Sca1^−^cKit^+^ populations, including CMPs, GMPs, and MEPs (Figure 3e). Consistently, qPCR analyses showed that there were decreases in IRF2 (Figure 3b) and IRF8 (Figure 3d) expression in Mysm1^−/−^Lin^−^ BM cells. Because IRF8 commits myeloid progenitors toward macrophages,^29^ we next isolated Lin^−^ cells from WT or Mysm1^−/−^ BM and examined their differentiation under either granulocyte- or macrophage-differentiation conditions. Our results show that Mysm1^−/−^Lin^−^ cells generated ~50% of the granulocytes and only ~25% of the macrophages that WT Lin^−^ cultures produced (Figure 3f). These results suggest that macrophage differentiation is more severely impaired than that of granulocytes, supporting that deficient IRF8 expression occurs in Mysm1^−/−^ Lin^−^ BM cells. To further confirm the deficient IRF2 expression in Mysm1^−/−^ BM, 5- to 6-week-old WT or Mysm1^−/−^ mice were injected with the type-I IFN inducer, Poly(I:C). We found that Sca1 expression was induced at 48 h, and then greatly recovered at 96 h post injection in the Lin^−^ BM cells from WT mice, presumably because IFN signaling repressors were expressed.^30^ However, in Lin^−^ BM cells from Mysm1^−/−^ mice, Sca1 was more strongly induced at 48 h, and the expression continued to increase from 48 to 96 h post injection, albeit at a lower rate (Supplementary Figure 2). These results provide evidence that IRF2 is downregulated in Mysm1^−/−^ mice.

**Figure 4:**
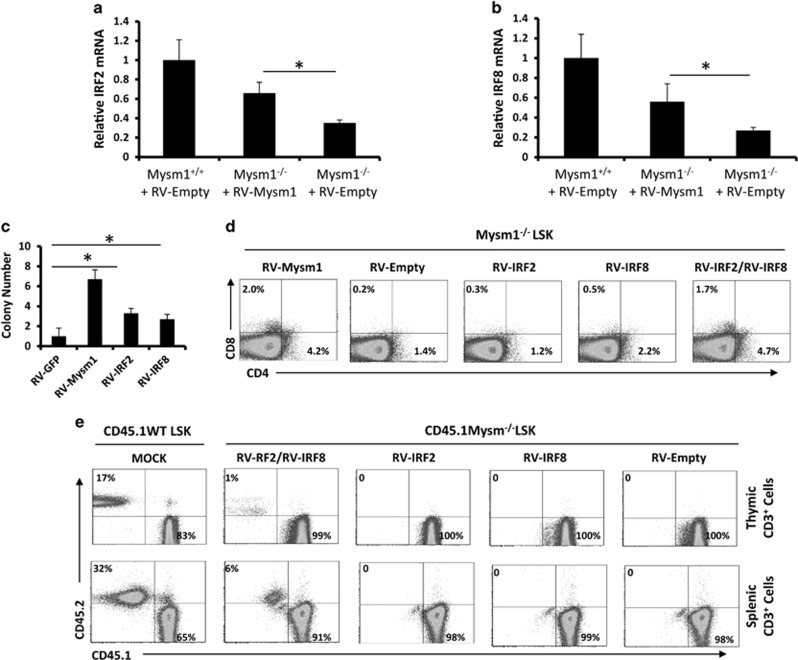
Co-expression of IRF2 and IRF8 partially rescue T-cell differentiation in Mysm1^−/−^ LSKs. (**a** and **b**) LSK cells were sorted from 8- to 10-week-old WT or Mysm1^−/−^ mice (*n*=4), and transduced with RV-empty or RV-Mysm1. Cellular RNA was extracted for determination of IRF2 expression (**a**) and IRF8 expression (**b**) at the mRNA level by qPCR. Bar graphs show means of three experiments±S.D. *P<0.05. (**c**) LSKs sorted from 8- to 10-week-old Mysm1^−/−^ mice (*n*=4) were transduced with RV-empty, RV-Mysm1, RV-IRF2, or RV-IRF8. The transduced LSKs were cultured on OP-9DL monolayers in RPMI-1640 supplemented with IL-7/Flt3L for 2 weeks. T-cell colonies were counted from a 96-well plate. Bar graphs show means of three experiments±S.D. *P<0.05. (**d**) Sorted LSKs (*n*=4) were transduced with RV-Mysm1, RV-empty, RV-IRF2, RV-IRF8, or RV-IRF2/RV-IRF8. The transduced LSKs were cultured on OP-9DL monolayers in RPMI-1640 supplemented with IL-7/Flt3L for 2 weeks. The T-cell markers CD4 or CD8 were analyzed by FACS. (**e**) LSKs (CD45.2) were isolated from WT or Mysm1^−/−^ mice (*n*=8–10). Mysm1^−/−^ LSKs were transduced with RV-empty, RV-IRF2, RV-IRF8, or RV-IRF2+RV-IRF8. The WT LSKs and the RV-transduced Mysm1^−/−^ LSKs (0.5 million) were respectively mixed with WT BM cells (CD45.1, 1 million) and i.v. injected into WT mice (CD45.1). Six weeks later, thymocytes and splenocytes were isolated and analyzed via FACS to determine the differentiation of LSK into T cells in recipient mice (*n*=4). All the experiments were repeated at least two times

Next, we performed *in vitro* rescue assays in which Mysm1^−/−^ LSKs were transduced with RV-Msym1, RV-IRF2, RV-IRF8, or an empty RV. qPCR analysis revealed that RV-Mysm1 increased expression of IRF2 (Figure 4a) and IRF8 (Figure 4b) in Mysm1^−/−^ LSKs 72 h after transduction. A colony forming assay further revealed that RV-Mysm1 transduction enhanced cell colony numbers by ~6.5-fold, RV-IRF2 by ~3.3-fold, and RV-IRF8 by ~2.7-fold compared with those of empty RV (Figure 4c). However, FACS analyses only showed that co-transduction with RV-IRF2 and RV-IRF8 significantly increased T-cell marker expression (prominently CD8) in Mysm1^−/−^ LSKs (Figure 4d). Furthermore, a competitive transplantation assay as described in Figure 3c was performed to study the transduced Mysm1^−/−^ LSKs. The results showed that small percentages of thymic and splenic T cells were produced from the transplanted, RV-IRF2/RV-IRF8 co-transduced Mysm1^−/−^ LSKs (Figure 4e), suggesting a synergistic role for IRF2 and IRF8 in T-cell differentiation.

**Figure 5:**
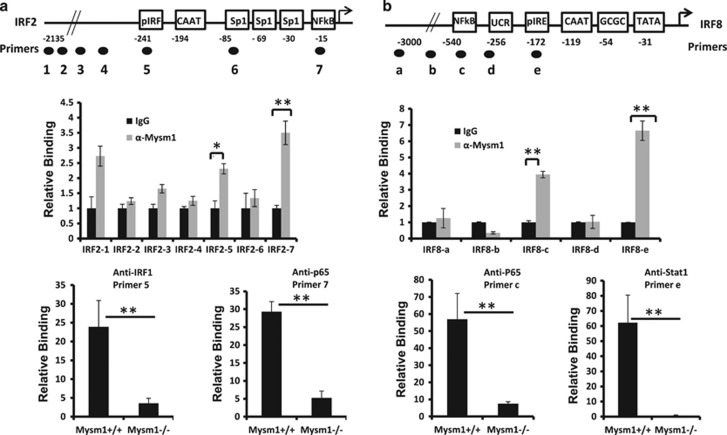
Mysm1 binds to the promoters of IRF2 and IRF8. (**a**) The site-specific primers were designed from the promoter region of IRF2 (*top*). ChIP assay analyzed the binding of Mysm1 to the promoter regions of IRF2 in the Lin^−^ BM cells from WT mice (*n*=4). The prepared chromatin was immunoprecipitated with either anti-Mysm1 antibody or control IgG, and co-precipitated DNA was eluted and subjected to qPCR analysis using the designed IRF2 primers (*middle*). ChIP assay analyzed recruitment of IRF1 or NF-kBp65 to the promoter region of IRF2 in the Lin^−^ BM cells from WT or Mysm1^−/−^ mice (*n*=4). The prepared chromatin from the Lin^−^ BM cells of WT or Mysm1^−/−^ mice was immunoprecipitated with anti-IRF1, anti-NF-kBp65, or control IgG antibodies, and precipitated DNA was eluted and subjected to qPCR analysis using the designed primers corresponding to the binding sites of IRF and NF-kBp65 in the IRF2 gene loci (*lower*). (**b**) The site-specific primers were designed from the promoter region of IRF8 (*top*). ChIP assay analyzed recruitment of Mysm1 to the promoter region of IRF8 in the Lin^−^ BM cells from WT mice (*n*=4) (*middle*). ChIP assay analyzed recruitment of Stat1 or NF-kBp65 to the promoter regions of IRF8 gene loci in the Lin^−^ BM cells from WT or Mysm1^−/−^ mice (*n*=4) by using anti-Stat1, anti-NF-kBp65, or control IgG to precipitate chromatin (*lower*). Data are representative of three independent experiments. Bar graphs show means of three experiments±S.D. *P<0.05; **P<0.01

Given that Mysm1 de-represses gene transcription via binding to the promoter regions for histone modifications, therefore recruiting key transcription factors to these regions,^13, 14, 16, 17^ we synthesized the promoter site-specific primers for IRF2 (Figure 5a*, top*) and IRF8 (Figure 5b, *top*). We then tested Mysm1 binding to the promoter regions of IRF2 and IRF8 by analyzing WT (not Mysm1^−/−^) Lin^−^ BM cells via a ChIP assay after *in vitro* stimulation with Poly(I:C) or IFN-γ. qPCR analysis was performed using the promoter-specific primers and revealed that immunoprecipitation of anti-Mysm1 effectively enriched DNA fragments that contained IRF and NF-kB binding sites in the IRF2 promoter^31, 32^ (Figure 5a, *middle*) and DNA fragments that contained the interferon regulatory element (IRE) or NF-kB binding sites in the IRF8 promoter (Figure 5b, *middle*).^33^ As controls, the promoter-specific primers of Bcl-2 or Bax failed to detect any significant Mysm1 binding (Supplementary Figure 3). These results indicate that Mysm1 specifically localizes to the promoter regions of the IRFs. We next used anti-IRF1, anti-Stat1, or anti-NF-kB (p65) antibodies to analyze WT or Mysm1^−/−^ Lin^−^ BM cells. As expected, anti-IRF1 and anti-NF-kB effectively precipitated the cognate DNA fragments of the IRF2 promoter from WT Lin^−^ BM cells but not from Mysm1^−/−^ Lin^−^ BM cells (Figure 5a, *lower*), and anti-Stat1 and anti-NF-kB effectively precipitated DNA fragments of the IRF8 promoter from WT Lin^−^ BM cells but not from Mysm1^−/−^ Lin^−^ BM cells (Figure 5b, *lower*). Ultimately, we tested functionality of Mysm1 in activating transcription of IRF2 and IRF8 by luciferase reporter assay. The results showed that Mysm1 significantly enhances IRF2 and IRF8 promoter activity (−600 to +50) (Supplementary Figure 4). Together, these results suggest that Mysm1 is required for directing transcription factors to the promoter regions of IRF2 and IRF8 in HSCs.

### IRF2 and IRF8 reduction in Mysm1^−/−^ HSCs enhances p53 levels

**Figure 6:**
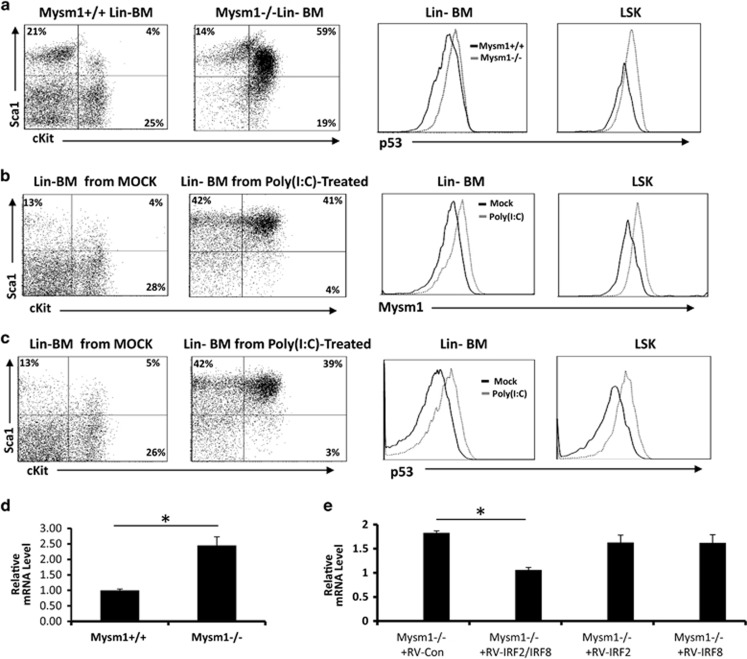
IRF2 and/or IRF8 suppresses p53 transcription in Mysm1^−/−^ HSCs. (**a**) BM cells from ~8-week-old WT or Mysm1^−/−^ mice (*n*=4) were analyzed by FACS for p53 protein levels in Lin^−^ BM cells or LSKs. (**b** and **c**) ~8-week-old normal mice were injected with Poly(I:C) (20 *μ*g/g body weight). Two days post injection, BM cells from Poly(I:C)-treated or non-treated mice (*n*=4) were analyzed by FACS for the protein levels of Mysm1 (**b**) or p53 (**c**) in Lin^−^ BM cells or LSKs. (**d**) Lin^−^ BM cells from ~8-week-old WT or Mysm1^−/−^ mice (*n*=4) were analyzed by qPCR for expression of p53 mRNA after *in vitro* stimulation with 100 ng of Poly(I:C) for 4 h. (**e**) Lin^−^ BM cells from ~8-week-old Mysm1^−/−^ mice (*n*=4) were transduced with the indicated RV vectors. Two days later, the transduced Mysm1^−/−^ Lin^−^ cells were sorted and the GFP^+^ cells were analyzed for expression of p53 mRNA by qPCR. Bar graphs show means of three experiments±S.D. *P<0.05

Nijnik *et al.*^15^ demonstrated that Mysm1^−/−^ mice have enhanced levels of p53, which is a central regulator of cellular stress responses. Belle *et al.*^34^ and Gatzka *et al.*^35^ independently generated Mysm1^−/−^p53^−/−^ mice that largely restored Mysm1 deficiency-associated defects. Moreover, Gatzka *et al.*^35^ revealed that increased p53 levels in Mysm1^−/−^ mice may result from the upregulation of p19^ARF^ that stabilizes p53 by blocking nucleo-cytoplasmic shuttling of Mdm2.^35^ However, Mysm1 is a H2ADUB that generally functions as a transcription activator. How the transcription activator deficiency leads to upregulation of p53 or p19^ARF^, therefore, remained elusive. To investigate the underlying mechanisms, we first confirmed that Mysm1^−/−^ mice had enhanced p53 levels in the Lin^−^ HSCs and LSKs as determined by ICS assays (Figure 6a). Given that p53 transcription is inducible by type-I IFN signaling,^36^ we analyzed Poly(I:C)-treated mice and found that Poly(I:C) injection enhanced expression of both p53 and Mysm1 in WT mice (Figures 6b and c). These results suggest that the reduction of the IFN signaling suppressors IRF2 and IRF8 may contribute to an enhanced p53 transcription in Mysm1^−/−^ mice. We therefore tested the p53 mRNA levels in WT and Mysm1^−/−^ Lin^−^ HSCs, and found that Mysm1^−/−^ Lin^−^ HSCs indeed expressed an enhanced level of p53 mRNA (~2.5-fold) compared with those of WT Lin^−^ HSCs after a short-term, low-dose Poly(I:C) treatment (Figure 6d). Hence, we isolated Mysm1^−/−^ Lin^−^ HSCs for transduction with RV-IRF2, RV-IRF8, or RV-IRF2+RV-IRF8. It was found that RV-IRF2/RV-IRF8 co-transduction significantly reduced p53 mRNA expression (Figure 6e), which provides evidence that Mysm1-mediated expression of IRF2 and IRF8 has a role in controlling p53 transcription in Lin^−^ HSCs.

### Mysm1 deficiency reduces thymocyte survival

**Figure 7:**
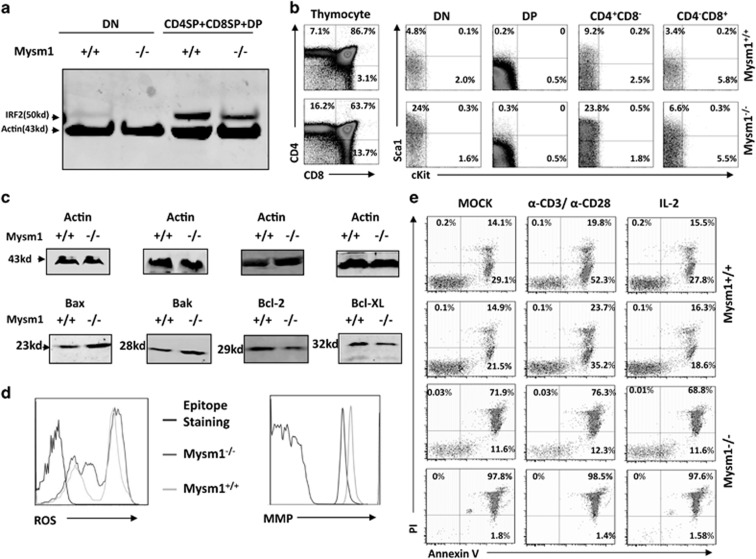
Thymocyte analysis in Mysm1^−/−^ mice. (**a**) Thymocytes were isolated from 8- to 10-week-old WT or Mysm1^−/−^ mice (*n*=4) for preparation of cell lysates. The cell lysates were fractionated by 4–12% SDS-PAGE and analyzed by WB to determine IRF2 expression. (**b**) Thymocytes were isolated from 8- to 10-week-old WT or Mysm1^−/−^ mice (*n*=4) and analyzed via immunostaining/FACS using anti-CD4, anti-CD8, and anti-Sca1 antibodies to determine Sca1 expression in the different stages of thymocyte development. (**c**) Thymocytes from 8- to 10-week-old WT or Mysm1^−/−^ mice (*n*=4) were used to prepare cell lysates. The cell lysates were analyzed via WB for detecting expression of pro-apoptotic proteins Bax/Bak and anti-apoptotic proteins Bcl-2/Bcl-x. (**d**) Thymocytes from 8- to 10-week-old WT or Mysm1^−/−^ mice (*n*=4) were analyzed for ROS levels (*left*) and for mitochondrial membrane potential (*right*). (**e**) The isolated thymoctyes were cultured overnight under the indicated conditions and analyzed via Annexin V/PI staining to define cell death. Data are representative of three independent experiments. DN, double-negative thymocyte; DP, double-positve thymocyte; SP, single-positive thymocyte

Previous studies detected an enhanced thymic p53 expression in Mysm1^−/−^ mice,^35^ which implies that Mysm1^−/−^ affects the thymus in addition to reducing input of CLPs from BM. Hence, we directly analyzed Mysm1^−/−^ thymocytes for IRF2 and IRF8 expression. Western blot (WB) analysis revealed that thymocytes from ~8-week-old Mysm1^−/−^ mice expressed lower levels of IRF2 relative to those of WT thymocytes (Figure 7a), whereas IRF8 expression in thymocytes from either Mysm1^−/−^ or WT mice was below detectable levels by WB. Consistently, immunostaining revealed increased levels of Sca1 in the CD4^−^CD8^−^, CD4^+^CD8^−^, and CD4^−^CD8^+^ thymocytes from these mice (Figure 7b). CD4^+^CD8^+^ thymocytes expressed increased levels of Sca1, but only occurred in ~16-week-old Mysm1^−/−^ mice. These older Mysm1^−/−^ mice exhibited a large reduction in CD4^+^CD8^+^ frequency (Supplementary Figure 5). Bamezai and Rock^37^ generated a strain of Sca1-transgenic mice using a T-cell lineage-specific promoter. They found that stage-specific loss of Sca1 expression is critical for normal thymic T-cell development, and that dysregulated Sca1 expression enhanced apoptosis of both Sca1-expressing and Sca1-non-expressing thymocytes through cell–cell adhesion mechanisms.^37, 38^ Thus, in addition to p53, dysregulated Sca1 likely contributes to thymocyte apoptosis in Mysm1^−/−^ mice. As a result, we directly analyzed the expression of pro- and anti-apoptotic proteins via WB. We found that freshly isolated thymocytes from Mysm1^−/−^ mice expressed elevated levels of pro-apoptotic proteins (Bax and Bak) and reduced levels of anti-apoptotic proteins (Bcl-2 and Bcl-x) relative to those of WT thymocytes (Figure 7c). The Mysm1^−/−^ thymocytes also produced enhanced levels of reactive oxygen species (ROS), but reduced the mitochondrial membrane potential (Figure 7d). Additionally, apoptosis analysis revealed that Mysm1^−/−^ thymocytes had much higher levels of cell death compared with WT thymocytes after overnight culture (Figure 7e).

**Figure 8:**
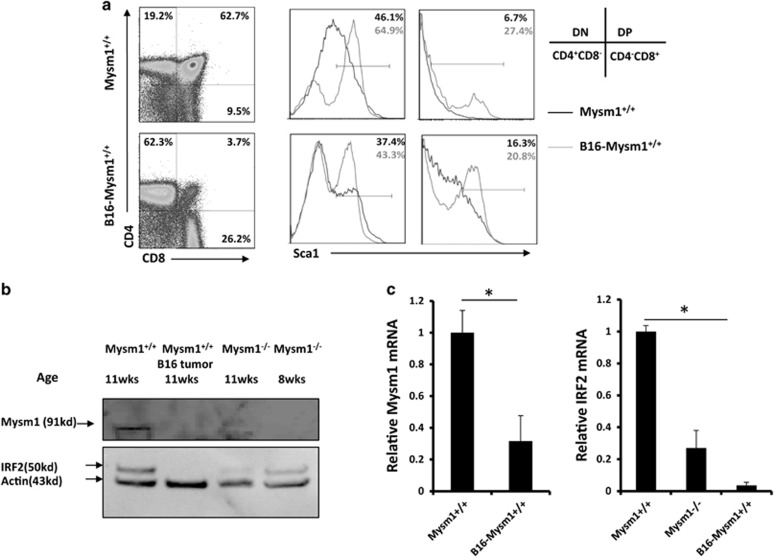
Thymocytes were analyzed in late-stage tumor-bearing mice. (**a**) WT mice (~8-week-old) received s.c. injection of melanoma B16 cells (1 × 10^6^ per mouse, *n*=4). Three weeks later, the B16-bearing mice in parallel with normal WT mice of the same age (~11 weeks old, *n*=4) were euthanized and analyzed for Sca1 expression in the different thymocytes stages. (**b**) Thymocytes were isolated from the B16 tumor-bearing mice, normal WT mice, or Mysm1^−/−^ mice (*n*=4) at the indicated ages for preparation of cell lysates. The prepared cell lysates were analyzed by WB for expression of IRF2 or Mysm1. (**c**) Total RNA was extracted from thymocytes isolated from euthanized mice and analyzed for the expression of Mysm1 (*left*) and IRF2 (*right*) by qPCR. All data are representative of at least three independent experiments. Bar graphs show means of three experiments±S.D. *P<0.01

The above results indicate that Mysm1 loss directly depletes thymocytes, which can lead to thymus atrophy. Thymus atrophy is a pathophysiological condition found in advanced ages and late-stage cancers. B16 melanoma-bearing mice show severe thymus atrophy at late tumor stages.^39^ Thus, we assessed HSC status in B16 tumor-bearing mice following B16 challenge. We observed no significant differences in the frequency of LSKs or in the expressions of IL-7R, Flt3, IRF2, or IRF8 between WT mice and late-stage cancer-bearing mice (Supplementary Figure 6A–C). We further analyzed the thymi of the B16 tumor-bearing mice via FACS. Results showed that only the CD4^−^CD8^−^ thymocytes had enhanced Sca1 expression at 14 days post tumor challenge (Supplementary Figure 7), but all four stages of thymocytes (CD4^−^CD8^−^, CD4^+^CD8^+^, CD4^−^CD8^+^, and CD4^+^CD8^−^) had drastically enhanced Sca1 expression at 21 days post challenge (Figure 8a). At that time point, the frequency of CD4^+^CD8^+^ thymocytes dropped to only ~3%, resembling the phenotype seen in ~16-week-old Mysm1^−/−^ mice (Supplementary Figure 5). WB assay showed that IRF2 expression did not significantly change at 14 days post challenge (*data not shown*), but almost disappeared by 21 days post challenge (Figure 8b). This was in agreement with qPCR results that showed a dramatic reduction in IRF2 at 21 days post tumor challenge (Figure 8c). Both WB and qPCR results showed that Mysm1 expression was drastically reduced at 21 days post challenge (Figures 8b and d). This suggests that the late-stage tumors may induce Mysm1 downregulation that contributes to thymus atrophy. The late-stage tumor-mediated change of peripheral environment has a limited influence on HSCs in BM. Tumor migration into BM was not detected at day 21 post inoculation (data not shown).

## Discussion

Mysm1 suppresses IFN-I signaling trough inactivation of TRAF3/TRAF6 complexes to protect against sepsis.^40^ The present study revealed that Mysm1 is required for expression of IRF2 and IRF8 in preserving self-renewal of HSC^23, 24, 25^ and in governing lineage commitment of HSC.^26, 27^ IRF2 functions as a suppressor of IFN-I signaling by occupying the IRF consensus site on target genes and subsequently preventing DNA binding by IRF1,^41^ which has an important role in maintaining quiescent HSC pool. Mysm1^−/−^ mice had enhanced HSC proliferation that led to a large dysfunctional LSK population. The reduced expression of IRF2 in Mysm1^−/−^ HSCs at least partially contributed to generation of the dysfunctional LSK population. Thus, we demonstrate that Mysm1 is required for expression of IRF2 in the maintenance of the HSC pool. IRF8, another IRF member, selectively represses ISRE-containing promoters and inhibits IFN-I signaling in certain situations.^42^ IRF8 regulates the commitment and differentiation of B cells and macrophages,^29, 43, 44^ and as such, has a critical role in the generation of these cells. Although IRF8 has not been reported to be involved in the maintenance of the HSC pool, some experimental data suggest that IRF8^−/−^ HSCs express increased levels of cKit,^26^ and high cKit expression identifies HSCs with impaired self-renewal and long-term reconstitution potential.^45^ Thus, the present study also implies that Mysm1-mediated IRF8 expression has a role in maintaining the quiescent HSC pool by limiting the generation of cKit^+^ HSCs. Given that IRF8 has a very weak DNA binding affinity,^46^ which is dramatically increased following interaction with IRF-1 and IRF2,^47, 48^ we suspect that IRF8 limits cKit expression likely by interacting with IRF2 since Mysm1^−/−^ mice show much higher cKit expression than that of IRF8^−/−^ mice. Moreover, the IRF2/IRF8 interaction may also strengthen control of IFN signaling.

Recent studies indicated that Mysm1^−/−^ mice expressed enhanced levels of p53 and Mysm1^−/−^-associated defects were largely restored by co-knockout of p53. Further studies revealed that Mysm1 suppressed p53-target gene Bbc3/PUMA for HSC maintenance^49^ and Mysm1^−/−^ enhanced p53 levels by upregulation of p19^ARF^. As Mysm1 generally functions as a transcription activator, it is rational to postulate that Mysm1 suppresses p53 or p19^ARF^ likely by activating expression of transcription repressors. Early studies demonstrated that p53 was induced by IFN-I signaling.^36^ Here, we observed enhanced p53 levels in Poly(I:C)-treated mice that paralleled increases in Mysm1 expression, and that there was enhanced p53 transcription in Mysm1^−/−^ HSCs. Forced expression of IRF2 and IRF8 significantly reduced p53 mRNA levels in Mysm1^−/−^ HSCs, suggesting that IRF2 and IRF8 have a direct or indirect role in suppressing p53 transcription in mouse HSCs. Gatzka *et al.* revealed that Mysm1^−/−^ mice had a dramatic increase in p19^ARF^ that stabilizes p53 by blocking nucleo-cytoplasmic shuttling of Mdm2. These studies altogether suggested that Mysm1 uses multiple mechanisms to regulate p53, and that IRF2/IRF8 may only contribute to p53 transcription control. This may explain why forced co-expression of IRF2/IRF8 only moderately recovered T-cell differentiation in the rescue assays, though the relatively low transduction efficiency of recombinant retroviruses may have been a limiting factor.

The study revealed that Mysm1^−/−^ HSCs expressed lower levels of IL-7R and Flt3, which may explain why Mysm1^−/−^ mice with a large LSK population in the BM develop lymphocytopenia. The study defined that Mysm1^−/−^ negatively impacts on turnover (differentiation/proliferation/death) of lymphoid progenitor cells and thymocytes, implying that Mysm1 may function as a general (not specific) regulator for T-cell lineage development. This is consistent with our previous finding that Mysm1 was not required for transcription of several T cell-specific differentiation factors,^13^ but was absolutely critical for the expression of several B cell- and NK cell-specific differentiation factors.^13, 14^ These results may explain why the enhanced frequencies of T cells are observed in Mysm1^−/−^ mice.

In conclusion, we provide strong evidence that Mysm1 is required for the expression of IRFs that have roles in HSC maintenance and thymocyte development.

## Materials and Methods

### Mice

Mysm1^−/−^ mice were generated as previously described,^13^ and maintained in a pathogen-free barrier facility. All experiments were performed in accordance with the University of Southern California Institutional Animal Care and Use Committee.

### FACS and cell sorting

Sample preparation, cytometric analyses, and cell sorting were performed as described.^50, 51^ Cells were flushed out of BM, and single-cell suspensions from the thymi and spleens were prepared. Cells were stained for 20 min at 4 °C with CD16/CD32 Fc-blocking antibody (2.4G2) in FACS buffer unless otherwise indicated, followed by incubation with a fluorophore-conjugated antibody. For each stain, at least 200 000 events were counted for analysis. Data were collected on a FACSCanto II (BD Biosciences, San Jose, CA, USA) and were analyzed with FlowJo software (TreeStar, Ashland, OR, USA). For cell progenitor population sorting, cells from BM were first depleted of mature hematopoietic cells with a lineage cell depletion kit (Miltenyi Biotec, Bergisch Gladbach, Germany), and then isolated with the FACSAria cell sorter (BD Biosciences).

### Cell proliferation and cell-cycle studies

*In vivo* incorporation of BrdU into LSK cells was assessed by APC or FITC BrdU FACS kit (BD Biosciences). Mice were intraperitoneally (i.p.) injected with BrdU (2 mg/mouse) for 5 days and then euthanized. BM cells were prepared and stained with fluorophore-conjugated antibodies and analyzed by FACS. For cell-cycle analysis, mice received a single i.p. injection of BrdU (2 mg/mouse). One hour later, mice were euthanized, and BM cells were stained and analyzed by FACS.

### Pyronin and Hoechst staining

BM cells were incubated in PBS containing 2% fetal calf serum and 10 *μ*M Hoechst 33342 (Molecular Probes, Carlsbad, CA, USA) for 30 min at 37 °C, then washed and resuspended in PBS supplemented with 10% FBS, 10 *μ*M Hoechst 33342, and pyronin Y (1 *μ*g/ml, Sigma-Aldrich, St. Louis, MO, USA). Cells were incubated for an additional 15 min at 37 °C, and then washed and stained for FACS.

### CHIP assay

Chromatin was immunoprecipitated using a ChIP kit according to the manufacturer's instructions (Cell Signaling Technology, Danvers, MA, USA). Cells were crosslinked with formaldehyde, and then chromatin was isolated, digested by micrococcal nuclease, sonicated, and immunoprecipitated with target antibodies. The immunoprecipitated chromatin was then eluted with ChIP elution buffer. DNA fragments were released by treatment with RNase A and proteinase K at 65 °C for 2 h. The released DNA fragments were purified with columns and amplified by site-specific primers by qPCR according to the manufacturer's protocol (Illumina, San Diego, CA, USA).

### Tumor growth

Eight-week-old C57BL/6 mice were subcutaneously inoculated with 1 million B16 cells per mouse. Around 14 or 21 days after tumor inoculation, the tumor-bearing mice were euthanized and thymus harvested from each mouse and analyzed for Mysm1 and IRF2 expression and frequency of different T cells.

### Statistics

Groups of 4–8 mice were used for statistical analysis. *P-*values were calculated with Student's *t*-tests.

## Abbreviations

HSCs: hematopoietic stem cells
Gfi-1: growth factor independence*-*1
LSKs: Lin^−^Sca1^+^cKit^+^ cell population
WT: wild type
IRF: interferon regulatory factor
IFN-I: type-I interferon
PRC1: polycomb repressor complex 1
Mysm1: Myb-like, SWIRM, and MPN domains containing protein 1
H2ADUB: H2A deubiquitinase
AR: androgen receptor
DC: dendritic cell
LNs: lymph nodes
BM: bone marrow
ETPs: early T lineage progenitors
CLPs: common lymphoid progenitors
FACS: flow cytometry
ICS: Intracellular staining
5-FU: 5-Fluorouracil
qPCR: quantitative PCR
CMPs: common myeloid progenitors
GMPs: granulocyte/macrophage progenitors
MEPs: megakaryocyte-erythroid progenitors
RV: retroviral vector
ChIP: chromatin immunoprecipitation
Mdm2: mouse double minute 2 homolog
BrdU: 5-bromo-2-Deoxyuridine
ISRE: IFN-stimulated response element
TRAF: TNF receptor-associated factor
PUMA: p53 upregulated modulator of apoptosis
