# Supplementary material for: Mysm1 is required for interferon regulatory factor expression in maintaining HSC quiescence and thymocyte development
Source: Cell Death Dis. 2016 Jun 9;7(6):e2260–. doi: 10.1038/cddis.2016.162 (PMC5143390; doi:10.1038/cddis.2016.162)
Supplement: Supplementary Figure Legends [file cddis2016162x1.doc]

**Supplemental Figure Legends**

**sFig. 1. T cell development is defective in MYSM1^-/-^ mice. A.** FACS analyzed CD4^+^ and CD8^+^ T cells from spleen and inguinal lymph nodes (ILN) of 8-10 week-old wild type (WT) or Mysm1^-/-^ mice (n=4). B. Frequency and cell numbers of CD4^+^ and CD8^+^ T cells in spleen and ILN from the WT or Mysm1^-/-^ mice.  **C.** Thymus from 8-10 week-old WT or Mysm1^-/-^ mice were measured for size (**top**) and cell numbers (**lower**) (n=4). **D.**  FACS analyzed DN, DP, CD4^+^CD8^-^, and CD4^-^CD8^+^ T cells in the thymus (n=6). **E**. Frequency and cell numbers of DN, DP, CD4^+^CD8^-^, and CD4^-^CD8^+^ T cells in WT and Mysm1^-/-^ thymus. **F.** Thymocytes from 8-10 week-old WT or Mysm1^-/-^ mice were analyzed by FACS for the frequency of ETP (n=4). **G.**  BM cells from 8-10 week-old WT or Mysm1^-/-^ mice were enumerated (n=4). **H.** BM cells from the WT or Mysm1^-/-^ mice were analyzed by FACS for the frequency of CLP. The data were shown from one of three independent experiments. * *P* < 0.01, ** *P* < 0.05**.**

**sFig. 2. Mysm1^-/-^ HSCs are more sensitive to poly(I:C) stimulation.** 4-6 week-old WT or Mysm1^-/-^ mice were i.p injected with 10ug/(g body weight) of poly(I:C) (4 mice per group). BM cells were isolated from the euthanized mice at 48 hr or 96 hr post-injection and analyzed for Sca1 expression on the Lin^-^ BM by immune staining/FACS.

**sFig. 3. Mysm1 does not bind to the promoter of Bcl-2 or Bax. A.** The site-specific primers amplifying the putative ISREs were designed for the promoter region of mouse Bcl-2 or Bax. **B-C.**  ChIP assay analyzed the binding of Mysm1 to the promoter region of Bcl-2 or Bax in the Lin^-^ BM cells from WT mice (n=4). The prepared chromatin was immunoprecipitated with either anti-Mysm1 antibody or control IgG, and co-precipitated DNA was eluted and subjected to qPCR analysis using the designed Bcl-2 primers (**B**) or Bax primers (**C**). Data are representative of three independent experiments. Bar graphs show means of three experiments ± S.D.

**sFig.4.** **Mysm1 activates the promoter of IRF2 or IRF8.**  **A**. Schematic representation of the mouse IRF2 (**left**) and IRF8 (**right**) promoter/enhancer luciferase reporter constructs. **B.** Luciferase reporter assays were performed on Raw264.7 cells that were cotransfected with the indicated reporter plasmid pGL3-basic or pGL3-pIRF2 (100ng) and pMIG or pMIG-Mysm1 (1ug) (**left**), or with the indicated reporter plasmid pGL3-basic or pGL3-pIRF8 (100ng) and pMIG or pMIG-Mysm1 (1ug) (**right**). Data are presented as mean ± SD, and the results are representative of three independent experiments. **P* < 0.05.

**sFig. 5**. **Sca1 expression in thymocytes in the various ages of Mysm1^-/-^ mice**. Thymocytes were isolated from various ages of Mysm1^-/-^ mice (10-week-old, 13-week-old, or 16-week-old) or WT Mysm1^+/+^ mice (16 week-old) (n=4) and analyzed for expression of Sca1 and cKit at the various stages of thymocytes.

**sFig. 6. LSK differentiation in later-stage tumor-bearing mice.** **A&B**, 8-week-old WT mice were s.c. inoculated with B16 melanoma cells (1x10^6^). BM cells were isolated from the euthanized B16 tumor-bearing mice at day 21 post-B16 inoculation, and also from normal WT mice or Mysm1^-/-^ mice of the same ages (4/group). The isolated BM cells were analyzed via immune staining/FACS for frequency of LSK (**A**) and for expression of IL-7R or Flt3 in Lin^-^ BM (**B**). **C**. Lin^-^ BM cells were isolated from these mice and cellular RNA was extracted for analyzing expression of IRF2 and IRF8 at the mRNA level via qPCR. Bar graphs show means of three experiments ± S.D. * *P* < 0.05, ** *P* < 0.01.

**sFig. 7. Thymocytes express a higher level of Sca1 at the DN stage in the tumor-bearing mice.** Thymocytes isolated from melanoma B16 tumor-bearing mice (14 days post-inoculation), or normal WT mice or Mysm1^-/-^ mice of the same ages. The isolated thymocytes were analyzed for Sca1 expression at different stages.

**Supplemental Experimental Procedures**

***Mice***

Mysm1^-/-^ mice were generated as described previously (Jiang et al., 2011). Briefly, Mysm1 mRNA truncation-first floxed mice (Mysm1^tm1a/tm1a^) were crossed with MMTV-cre mice in the B6129F1 background or Tek-cre in the BL/6 background for complete deletion of the floxed Mysm1 exon without any possible transcriptional leakage of the splice acceptor-capture, and RNA poly(A) termination strategy designed in the Mysm1-targeted vector. MMTV-Cre mice and Tek-Cre have a widespread pattern of Cre expression in various cells including hematopoietic cells, B and T cells and their progenitors (Kasper et al., 2006; Kim et al., 2008). In all experiments, WT littermates (Mysm1^+/+^) were used for controls. Mice were maintained in a pathogen-free barrier facility, and all experiments were performed in accordance with the University of Southern California Institutional Animal Care and Use Committee.

***Treatment with 5-FU***

Mice were i.p. injected with varying doses of 5-FU (0, 30 mg, or 60 mg per kg body weight). Three days later, mice were euthanized and BM cells were harvested for analyzing LSK differentiation and cell death by immune staining.

***Intracytoplasmic staining (ICS)***

For detection of IRF2 or IRF8 by flow cytometry, BM cells were first pre-stained with surface markers then fixed and made permeable with a Phosflow kit (BD Biosciences). The cells were then stained with anti-IRF2 (C-19, Santa Cruz Biotechnology) or anti-IRF8 (C-19) followed with PE-conjugated 2^nd^ antibodies (Sigma).

***Immunoblot analysis and immunoprecipitation***

Immunoblot analyses were performed as described (Jiang et al. 2011). Primary antibodies were as follows: anti-Bcl-2 (610538), anti-Bcl-xl (556361), anti-Bax (554106), and anti-Bak (556396; all from BD Biosciences), anti-IRF2 (C-19), and anti-Mysm1 (Custom-made).

***Retrovirus Production and Transduction***

Retroviruses were produced by transient transfection of phoenix cells with retroviral vectors that encode Mysm1 (pMIG-Mysm1), IRF2 (pMIG-IRF2), or IRF8 (pMIG-IRF8) as described (Pongubala et al., 2008). For transduction, hematopoietic cells seeded in the RetroNectin (TaKaRa)-pretreated culture dishes were added with the harvested retroviral supernatants and centrifuged at 3,000 rpm for 90 minutes, and then incubated at 37° C in the presence of polybrene (4 μg/ml) for an additional 48 hours.

***RNA extraction and qPCR***

Freshly isolated BM cells were first depleted of mature hematopoietic cells with a lineage cell depletion kit (Miltenyi Biotec). Total RNA was isolated using an RNeasy Mini kit (Qiagen, Alameda, CA) from the isolated Lin^-^ BM cells. cDNA was synthesized with iScript Advanced cDNA Synthesis Kit (Bio-Rad, Hercules, CA). qPCR was performed in triplicate with a CFX96 PCR system and iTaq Universal SYBR Green Supermix according to the manufacturer’s instructions (Bio-Rad). Data were analyzed with PCR Array Data Analysis Template Excel downloaded from the webpage <http://www.sabiosciences.com/pcrarraydataanalysis.php>

For qPCR analysis of p53 mRNA, a pair primers are the 5’-forward: 5’-ggaaatttgtatcccgagtatctg-3’; and the 3’-reverse: 5’-agtcttccagtgtgatgatggtaa-3’.

***Bone marrow transplantation.***

Sorted LSK cells (1x10^5^) from WT or Mysm1^-/-^ mice (CD45.2) mixed with unfractionated BM cells (2x10^5^) from WT mice (CD45.1) were transplanted into lethally irradiated (9.5 Gy) WT C57BL/6 (CD45.1) mice through retro-orbital injection. PE-conjugated CD45.2 and FITC-conjugated CD45.1 (BD Biosciences) were used to determine the CD45.1- or CD45.2-derived population by flow cytometry analysis. Peripheral blood, spleen, and bone marrow were harvested and analyzed by flow cytometry.

***T cell colony forming assays.***

For T cell colony forming assays, BM cells from Mysm1*^-/-^* and WT mice were first enriched for hematopoietic progenitors by magnetic depletion (Miltenyi Biotec) with lineage-specific antibodies. LSK cells were sorted directly into 96-well plates containing pre-plated OP9-DL1 cells (ATCC). The cultures were supplemented with Flt3 ligand (10 ng/ml) and Il-7 (10 ng/ml). Cultures were substituted with fresh cytokines twice per week. After 7 to 10 days, wells were scored for the presence of B cell or T cell colonies.

***Macrophage/granulocyte colony forming assays.***

Lin^-^ BM cells were isolated as above described. 2× 10^4^ Lin^-^ cells were seeded onto methylcellulose medium (Methocult M3630 Enriched, Stem cell Technologies) in 35-mm-diameter petri dishes in duplicate, supplemented with 2 ng/ml or 20 ng/ml of M-CSF for macrophage differentiation or with 2 ng/ml or 20 ng/ml of IL-3 and G-CSF for granulocyte differentiation. Cultures were incubated at 37° C with 5% CO_2_ in a 100% humidified atmosphere for 7 days. Cell colonies were counted under microscope, and the mean number of colonies in the two dishes was calculated.

### Chromatin immunoprecipitation (CHIP).

Chromatin was immunoprecipitated according to the manufacturer’s instructions (Cell Signaling Technology). In brief, cells were crosslinked with formaldehyde. Chromatin was isolated, digested by micrococcal nuclease, sheared by sonication, and immunoprecipitated with antibodies. The immunoprecipitated chromatin was then eluted with ChIP elution buffer. DNA fragments were released by treatment of RNase A and then proteinase K at 65° C for 2 h. The released DNA fragments were purified with columns and amplified by site-specific primers by quantitative PCR (qPCR) according to the manufacturer’s protocol (Illumina). The following antibodies were used: anti-Mysm1 (custom-made); anti-Stat1 p84/p91 (M-22, Santa Cruz Biotechnology), anti-IRF1 (M-20, Santa Cruz Biotechnology); anti-NF-kB P65 (C-20, Santa Cruz Biotechnology), and anti-Sp1 (PEP2, Santa Cruz Biotechnology).

**Flow Cytometric Analyses and Cell Sorting**

Sample preparation, cytometric analysis, and sorting were performed as described previously. Cells were flushed out of BM and single cell suspensions from thymus and spleens were prepared. Cells were first stained for 20 min at 4° C with CD16/CD32 Fc-blocking antibody (2.4G2) in flow cytometry buffer unless indicated otherwise, followed by incubation with a ‘‘cocktail’’ of antibodies conjugated to fluorescein isothiocyanate (FITC), phycoerythrin (PE), peridinine chlorophyll protein complex-cyanine5.5 (PerCP-Cy5.5), phycoerythrin-indotricarbocyanine (PE-Cy7), allophycocyanin (APC), or allophycocyanin- indotricarbocyanine (APC-Cy7). For each staining, at least 200,000 events were collected for analysis. The following antibodies from BD Biosciences (San Diego, CA), eBioscience (San Diego, CA) and BioLegend (San Diego, CA) were used for flow cytometry: anti-mouse lineage cocktail (145-2C11, RB6-8C5, M1/70, RA3-6B2, Ter-119), anti-Sca1 (anti-Ly6A; D7), anti-CD117 (anti-cKit; 2B8), anti-CD127 (anti-IL-7R; A7R34), anti-CD150 (mShad150), anti-CD48 (HM48-1), anti-CD135 (Flt3; A2F10.1), anti-CD34 (RAM34), anti-B220 (RA3- 6B2), anti-CD3e (145-2C11), anti-CD4 (L3T4), anti-CD8a (53-6.7), anti-CD45.1 (A20), anti-CD45.2 (104), rat IgG2a k isotype (R35-95), rat IgG2b k isotype (A95-1), rat IgG1 k isotype (R3-34), rat IgG1 λ isotype (A110-1), hamster IgG1 k isotype (A19-3), and mouse IgG2a k isotype (G155-178). Data were collected on a FACSCanto II (BD Biosciences) and were analyzed with FlowJo software (TreeStar, Ashland, OR). For cell progenitor population sorting, cells from BM were first depleted of mature hematopoietic cells with a lineage cell depletion kit (Miltenyi Biotec, Bergisch Gladbach, Germany) and then isolated by FACSAria cell sorter (BD Biosciences).
