# Supplementary material for: Mysm1 is required for interferon regulatory factor expression in maintaining HSC quiescence and thymocyte development
Source: Cell Death Dis. 2016 Jun 9;7(6):e2260–. doi: 10.1038/cddis.2016.162 (PMC5143390; doi:10.1038/cddis.2016.162)
Supplement: Supplementary Figures [file cddis2016162x3.ppt]

## Slide 1
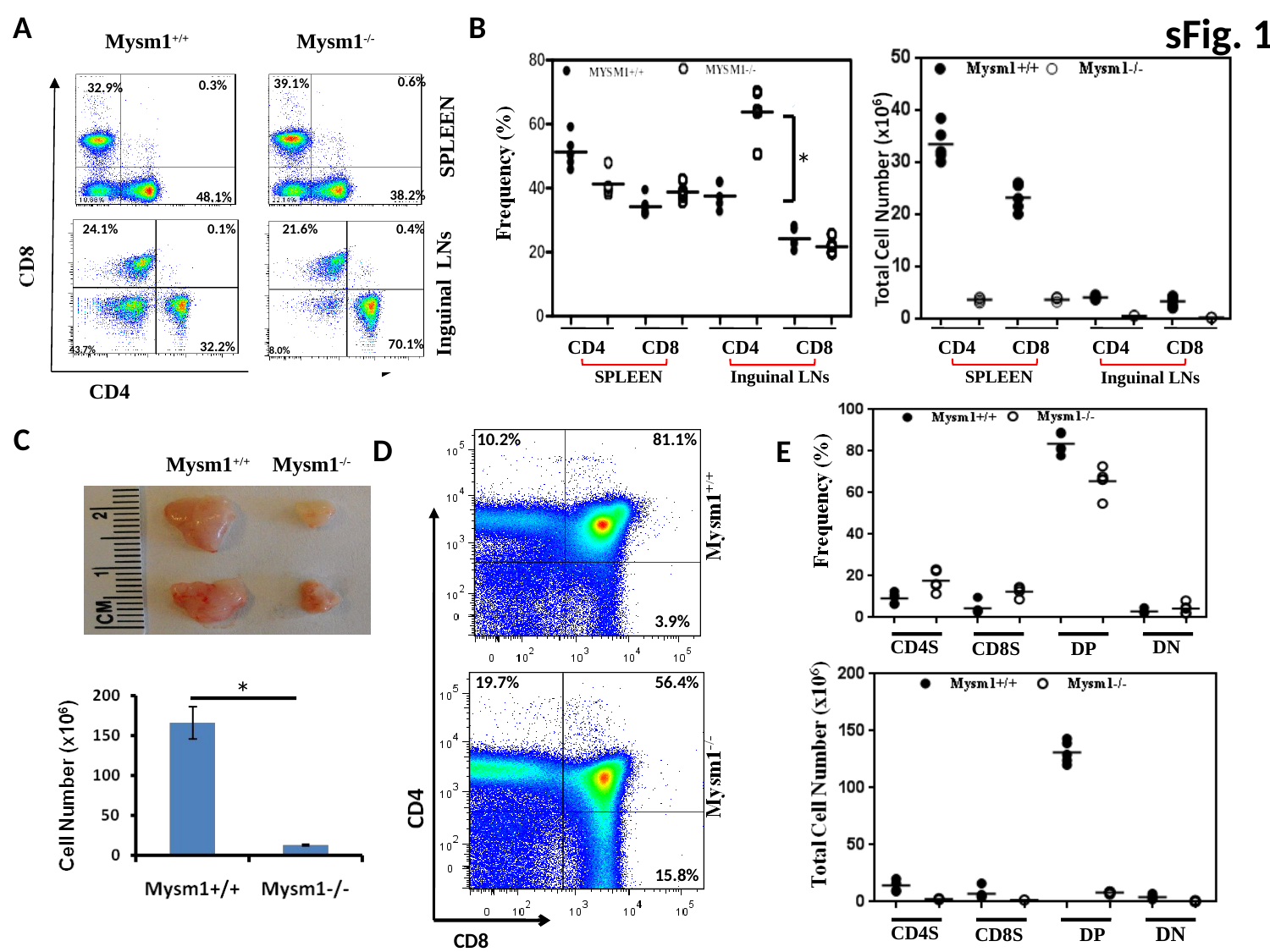

A
B
sFig. 1
 Mysm1+/+
 Mysm1-/-
0.6%
39.1%
0.3%
32.9%
SPLEEN
38.2%
48.1%
 21.6%
0.4%
70.1%
 8.0%
24.1%
0.1%
32.2%
43.7%
Inguinal LNs
CD4
*
 CD4
 CD8
CD8
 CD4
 CD8
CD4
CD8
CD4
SPLEEN
SPLEEN
 Inguinal LNs
 Inguinal LNs
C
10.2%
81.1%
D
E
 Mysm1+/+ Mysm1-/-
 CD4S
 CD8S
 DP
DN
3.9%
19.7%
56.4%
15.8%
*
 CD4S
DN
 CD8S
 DP
CD8

## Slide 2
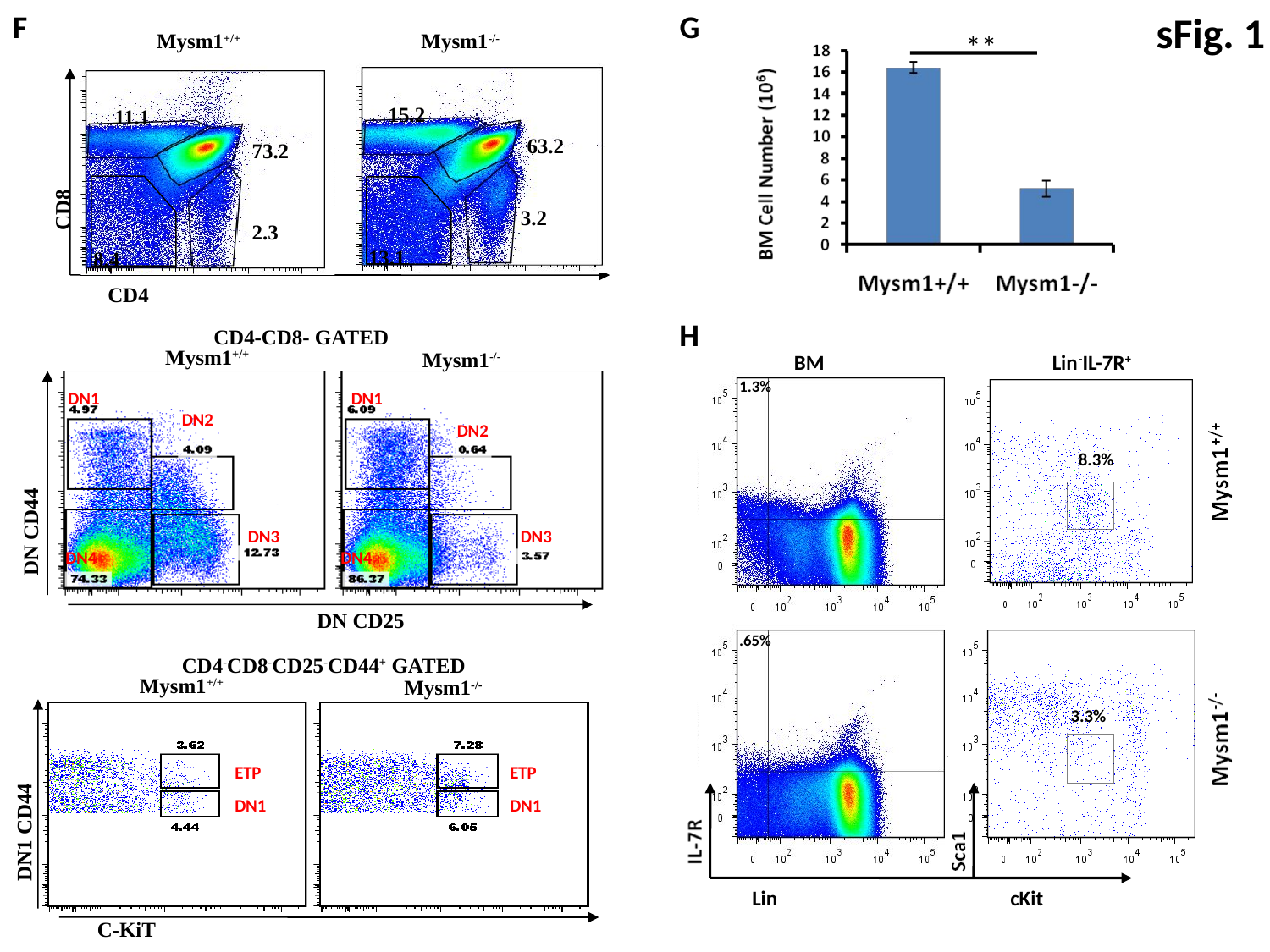

F
G
sFig. 1
Mysm1+/+
Mysm1-/-
**
15.2
63.2
3.2
13.1
11.1
73.2
CD8
2.3
8.4
CD4
H
CD4-CD8- GATED
Mysm1+/+
Mysm1-/-
DN1
DN1
DN2
DN2
 DN CD44
DN3
DN3
DN4
DN4
DN CD25
 BM Lin-IL-7R+
1.3%
8.3%
.65%
3.3%
Lin cKit
CD4-CD8-CD25-CD44+ GATED
Mysm1+/+
Mysm1-/-
ETP
ETP
DN1
DN1
 DN1 CD44
C-KiT

## Slide 3
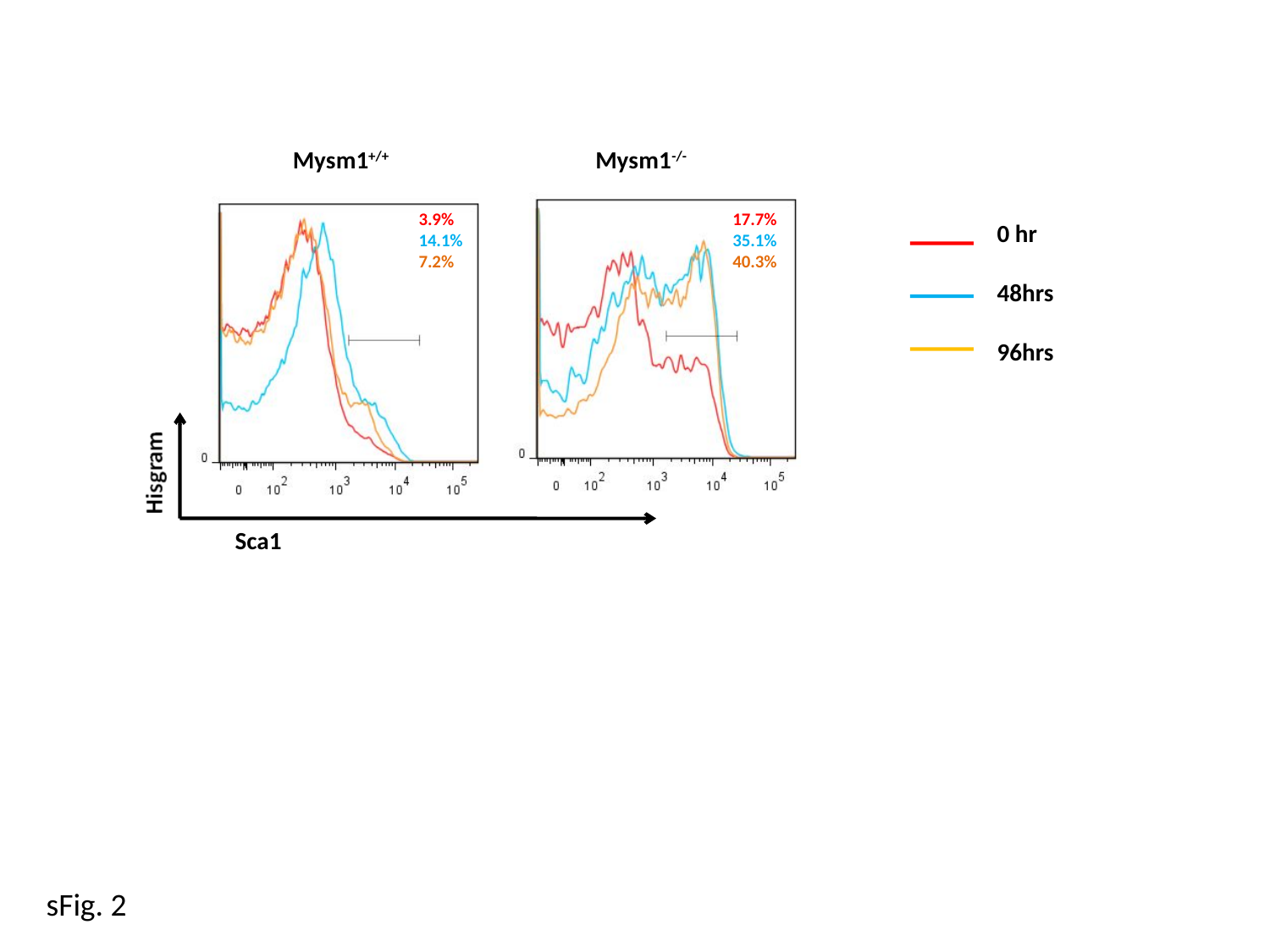

Mysm1+/+ Mysm1-/-
3.9%
14.1%
7.2%
17.7%
35.1%
40.3%
0 hr
48hrs
96hrs
Sca1
sFig. 2

## Slide 4
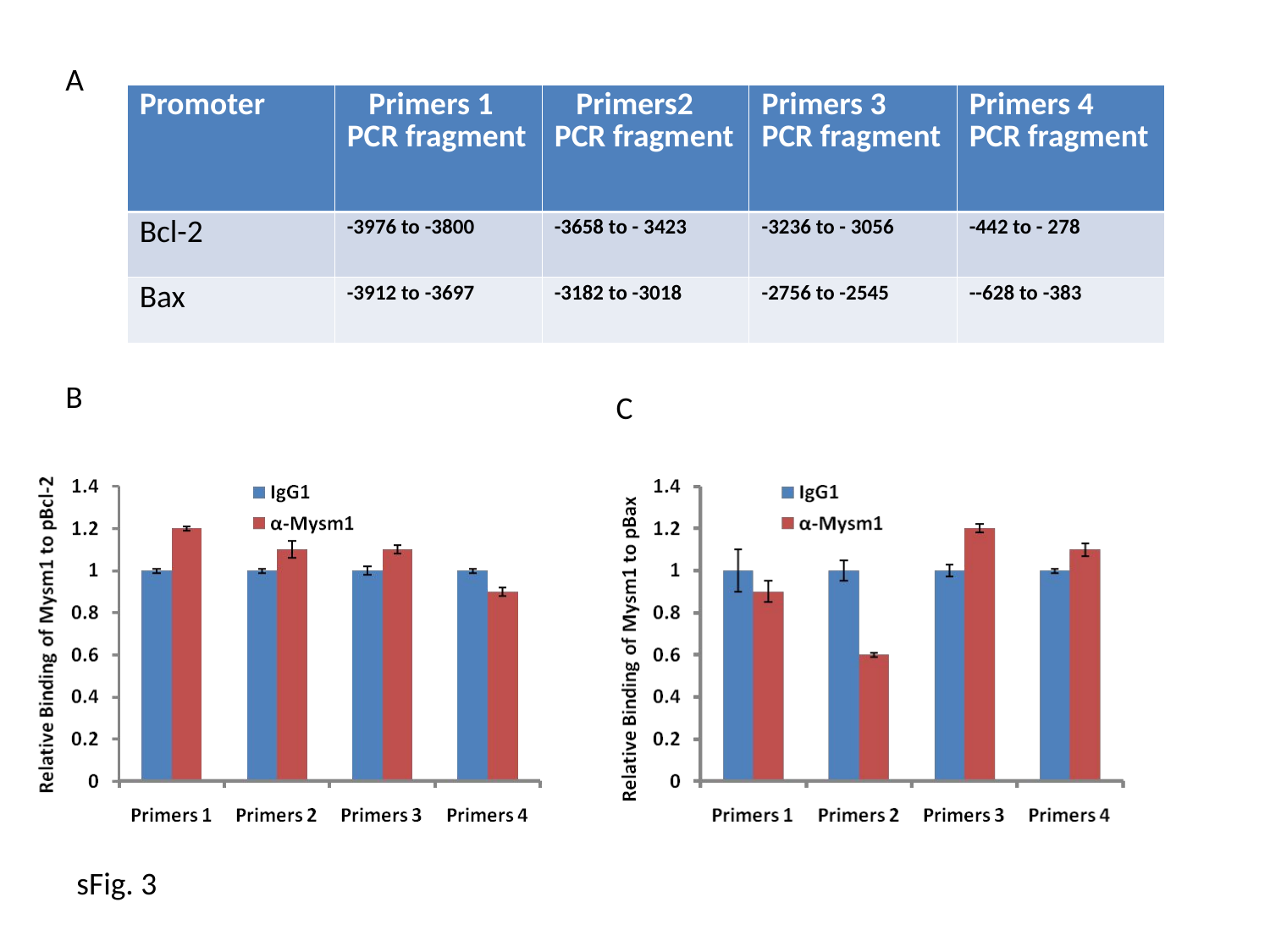

A
| Promoter | Primers 1 PCR fragment | Primers2 PCR fragment | Primers 3 PCR fragment | Primers 4 PCR fragment |
| --- | --- | --- | --- | --- |
| Bcl-2 | -3976 to -3800 | -3658 to - 3423 | -3236 to - 3056 | -442 to - 278 |
| Bax | -3912 to -3697 | -3182 to -3018 | -2756 to -2545 | --628 to -383 |
B
C
sFig. 3

## Slide 5
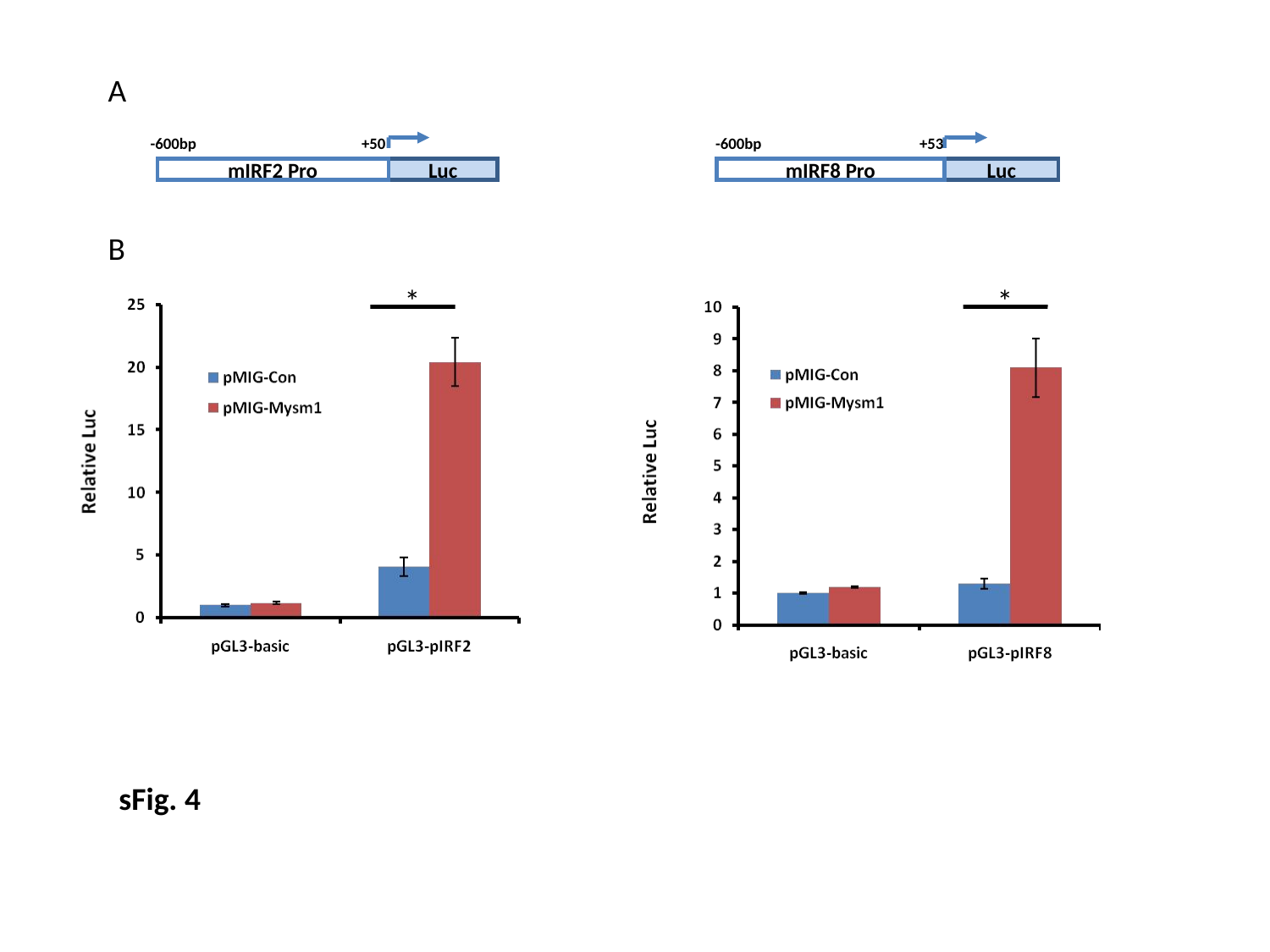

A
 -600bp +50
mIRF2 Pro
Luc
 -600bp +53
mIRF8 Pro
Luc
B
*
*
sFig. 4

## Slide 6
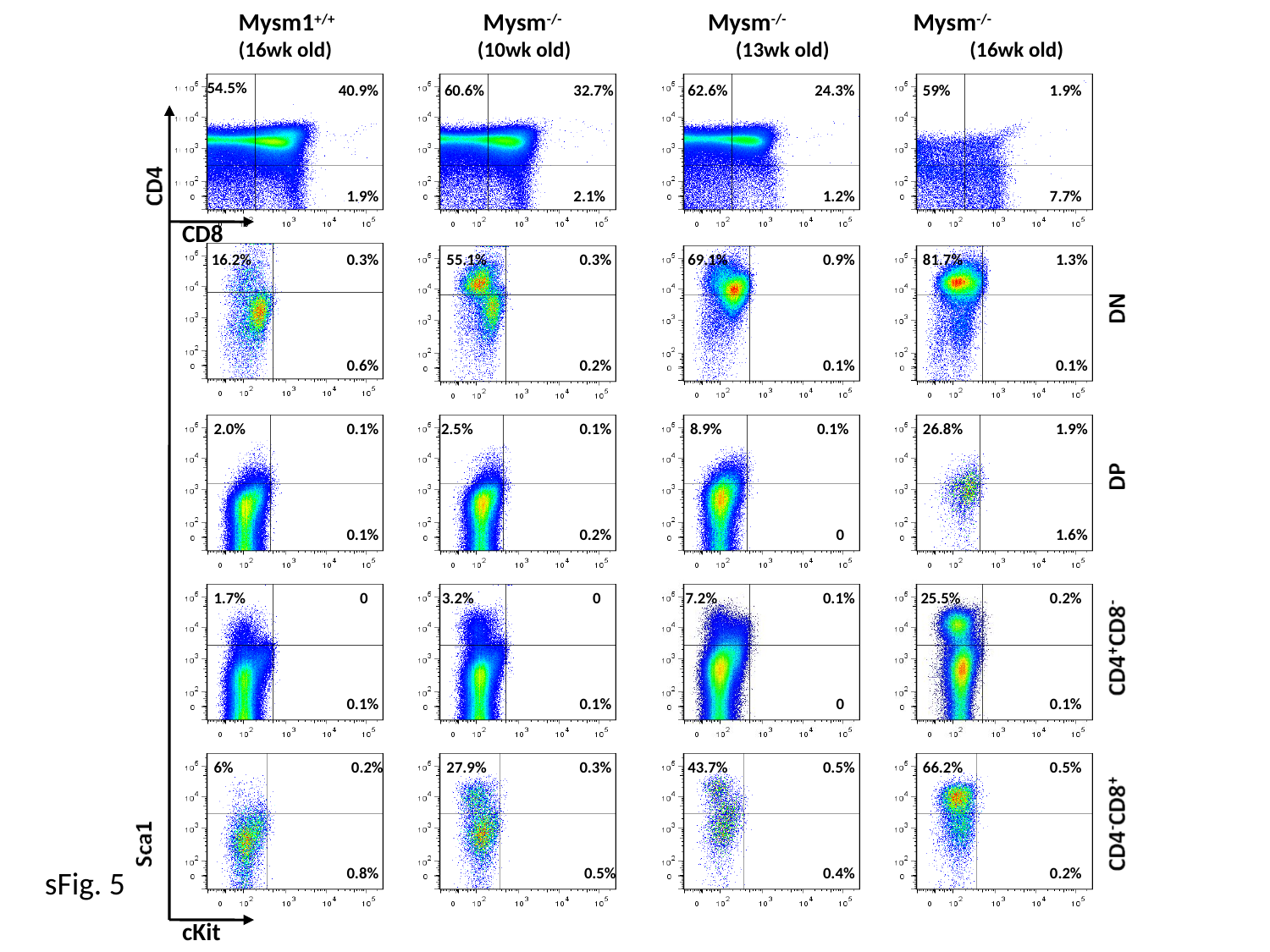

Mysm1+/+ Mysm-/- Mysm-/- Mysm-/-
(16wk old) (10wk old) (13wk old) (16wk old)
54.5%
40.9%
60.6%
32.7%
62.6%
24.3%
59%
1.9%
1.9%
2.1%
1.2%
7.7%
CD8
16.2%
0.3%
55.1%
0.3%
69.1%
0.9%
81.7%
1.3%
0.6%
0.2%
0.1%
0.1%
2.0%
0.1%
2.5%
0.1%
8.9%
0.1%
26.8%
1.9%
0.1%
0.2%
0
1.6%
1.7%
0
3.2%
0
7.2%
0.1%
25.5%
0.2%
0.1%
0.1%
0
0.1%
6%
0.2%
27.9%
0.3%
43.7%
0.5%
66.2%
0.5%
0.8%
0.5%
0.4%
0.2%
sFig. 5
cKit

## Slide 7
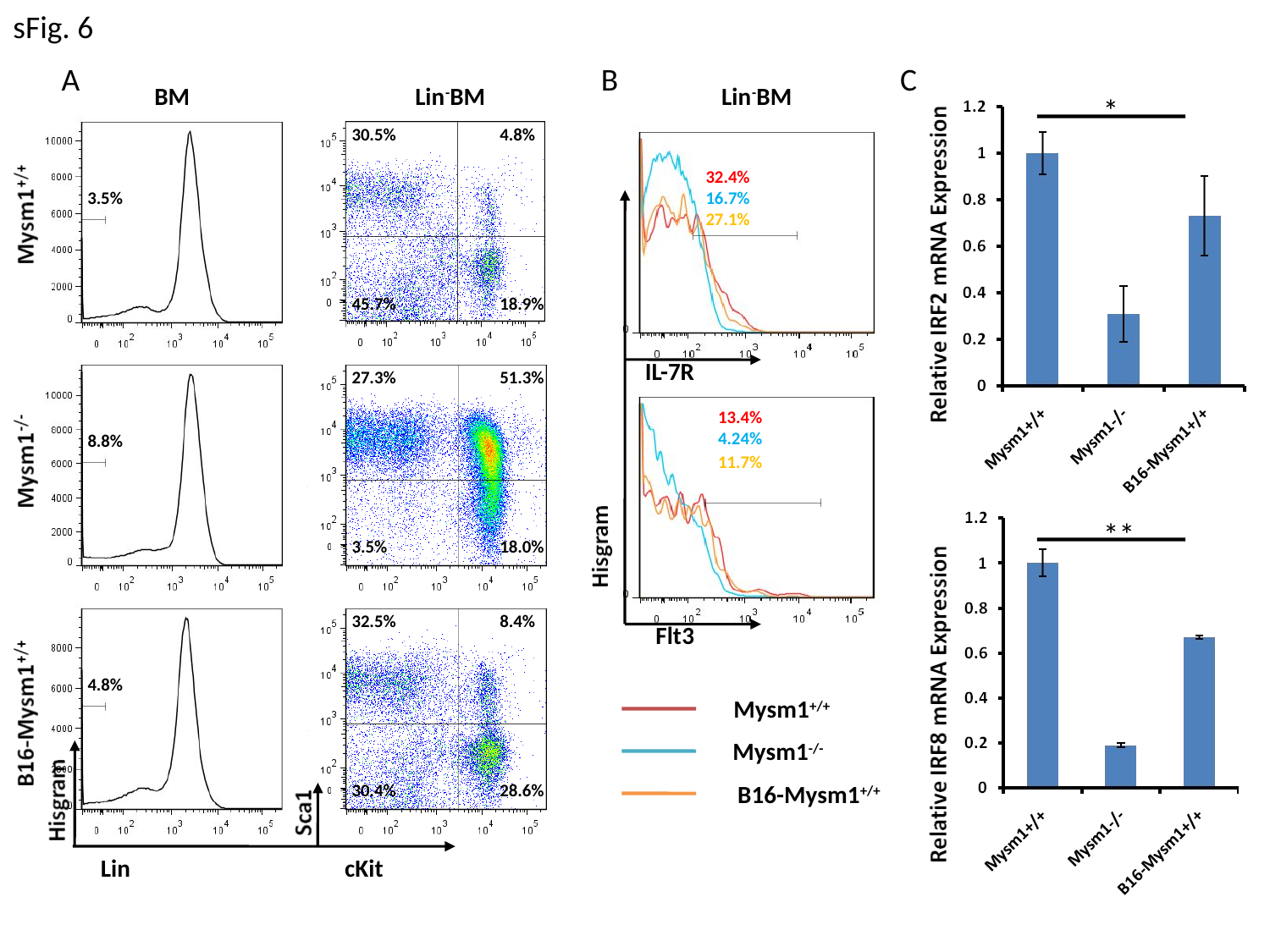

sFig. 6
A
B
C
BM Lin-BM Lin-BM
*
30.5%
4.8%
3.5%
45.7%
18.9%
27.3%
51.3%
8.8%
3.5%
18.0%
32.5%
8.4%
4.8%
30.4%
28.6%
Lin cKit
32.4%
16.7%
27.1%
IL-7R
13.4%
4.24%
11.7%
Flt3
Mysm1+/+
Mysm1-/-
B16-Mysm1+/+
**

## Slide 8
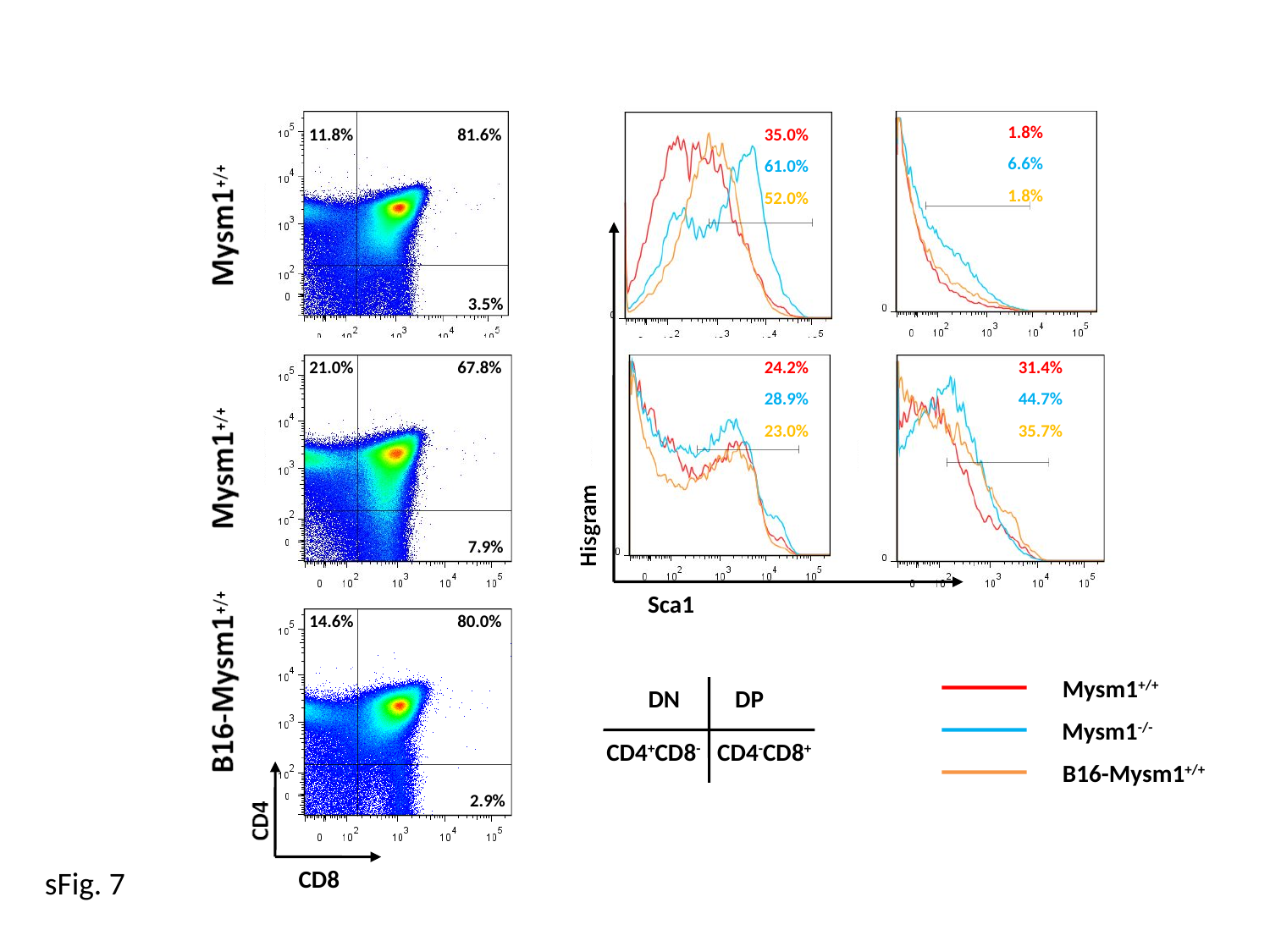

1.8%
11.8%
81.6%
35.0%
6.6%
61.0%
1.8%
52.0%
3.5%
21.0%
67.8%
24.2%
31.4%
28.9%
44.7%
23.0%
35.7%
7.9%
Sca1
14.6%
80.0%
Mysm1+/+
DN DP
Mysm1-/-
CD4+CD8-
CD4-CD8+
B16-Mysm1+/+
2.9%
sFig. 7
CD8
